# Reliability and validity of a questionnaire measuring knowledge, attitude and practice regarding “oil, salt and sugar” among canteen staff

**DOI:** 10.1038/s41598-023-47804-3

**Published:** 2023-11-22

**Authors:** Zhenhan Mo, Xiaofan Li, Yuting Zhai, Yanyan Men, Yuying Tang, Jiarui Qiao, Xue Jia, Yujie Huang, Baozhen Wang

**Affiliations:** 1 Department of Nutrition and Food Hygiene, School of Public Health, Cheeloo College of Medicine, Shandong University, 44, WenhuaXi Street, Jinan, 250012 Shandong Province People’s Republic of China; 2 Shandong First Medical University and Shandong Academy of Medical Sciences, Jinan, 250011 Shandong Province People’s Republic of China; 3 Zhaoyuan Center for Disease Control and Prevention, Zhaoyuan, 265400 Shandong People’s Republic of China; 4 Qilu Medical University, Zibo, 255300 Shandong People’s Republic of China

**Keywords:** Diseases, Health care

## Abstract

Excessive intake of oil, salt and sugar is closely associated with the prevalence of non-communicable chronic diseases (NCDs). Canteen staff’s knowledge, attitude and practice (KAP) about oil, salt and sugar directly affect the content in dishes and the consumers’ intake. However, no valid questionnaire is used to assess KAP among canteen staff about the “oil, salt and sugar”. Therefore, the present study aimed to establish and validate a questionnaire to evaluate the KAP of canteen staff about the “oil, salt and sugar”. This cross-sectional study was conducted among canteen staff randomly selected from three college canteens. Participants completed the questionnaire and retested it two weeks later. Internal and test–retest reliability were assessed using Cronbach’s α and Pearson correlation coefficients, respectively. Validity was assessed using the exploratory factor analysis. 100 participants finished the questionnaire, of which 66% were females with a mean age of 40.3 ± 10.5 years. The Cronbach’s α coefficients of the total questionnaire and Knowledge, Attitude and Practice dimensions were 0.822, 0.830, 0.752 and 0.700, respectively. The test–retest reliability coefficient was 0.968. In exploratory factor analysis, nine common factors were extracted, with 26 items, and the cumulative contribution rate was 70.9%. The questionnaire had a satisfactory property for measuring the KAP of the “oil, salt and sugar” among canteen staff in China.

## Introduction

Diet fat, salt and sugar as necessities of daily life, are important sources of essential macronutrients. However, excess intake of diet fat, salt and sugar is closely associated with the prevalence of non-communicable chronic diseases (NCDs) such as high blood pressure and cardiovascular diseases (CVDs)^1–4^. The literature based on population, published in Lancet^5–7^ demonstrated that decreased consumption of fiber-rich foods and increased consumption of high-fat, high-salt, high-sugar convenience foods and unhealthy lifestyle behaviors contribute to an increased risk of CVDs. This highlights the necessity for geographically targeted intervention strategies to ameliorate diet quality and mitigate the diet-related burden of CVDs.

For many industrialized countries, manufactured food and sugar-sweetened beverages are the primary sources of salt, saturated fat and sugar^8,9^. Taxation of unhealthy or luxury food and drink items and product reformulation targeted at reducing salt, saturated fat, and sugar have proven effective^10–12^. However, alternative strategies are needed for low-middle-income countries, such as China, where sodium and fat/oil intake are mainly from food prepared at home^13^. Population-based sodium and fat/oil reduction have emerged as effective strategies for managing hypertension, cardiovascular diseases, and obesity^13,14^.

College canteens are places for centralized dining. One crucial stage in developing healthy personal habits is during higher education, where the diets of college students who have left their family environment also undergo changes that can impact their health in the short and long term. However, the intake of oil, salt and sugar is related to the individual’s eating behavior and the canteen staff’s health literacy and cooking behavior, which can supply a healthy eating environment. Individual behavior change is more likely to be facilitated and sustained if the environment within which choices are made supports healthful food options^15^. Therefore, evaluating the KAP of canteen staff about oil, salt and sugar is very important.

To our knowledge, no questionnaire assesses canteen staff’s KAP of “oil, salt, and sugar”. Thus, the primary aim of this study was to establish and validate a questionnaire to evaluate the KAP of canteen staff about “oil, salt, and sugar”. In this initial study, we hypothesized that the KAP questionnaire would have good measurement properties (internal reliability, test–retest reliability and validity). As part of the nutrition and healthy canteen evaluation system, this questionnaire will help assess the KAP of canteen staff and guide future intervention studies and nutritional and healthy canteen construction.

## Methods

### Phase 1: Questionnaire development

The questionnaire was developed by reviewing the literature and group discussions. We comprehensively reviewed relevant government documents and the published literature to identify KAP questionnaire items about oil, salt, sugar, and health. After eight rounds of group discussions, the final questionnaire comprised four sections containing 76 items.

### Phase 2: Questionnaire validation

According to Walter^16^ and Mundfrom^17^, a sample size of at least 100 participants was considered adequate for test–retest reliability assessment (correlation coefficient) and factor analysis. Firstly, we randomly selected three college canteens in Shandong Province, and then 100 canteen staff as participants were selected in three different college canteens through cluster sampling. The inclusion criteria were adults aged ≥ 18 years and working experience ≥ 1 year who all signed an informed consent form and voluntarily participated in this study. Participants completed the paper questionnaire distributed by the researcher on-site and retested two weeks later. The questionnaire’s reliability and validity can be evaluated through the survey data.

### Statistical analysis

Statistical analysis was performed using SPSS Statistics software (version 24.0). Mean and standard deviations were calculated for continuous variables, and frequencies and percentages were calculated for categorical variables. Cronbach’s α was used to evaluate the internal consistency of the questionnaires, which was 0.7 or higher, indicating a satisfactory level^18^. Pearson’s correlation coefficient was used to calculate the test–retest reliability. As per Cade et al.,^19^ correlation coefficients were defined as 0.5 or greater, which indicated higher reliability. Exploratory factor analysis using the principal axis factoring method and varimax rotation determined the construct validity.

The Kaiser–Mayer–Olkin (KMO) measure was used to assess sample adequacy, and values of more than 0.5 showed that the data was suitable for factor analysis. We applied the principal component method and varimax rotation to extract items into factors. The maximum variance orthogonal rotation method is used, and the principle of eigenvalue > 1 is selected for extraction. A two-sided *p*-value < 0.05 was considered statistically significant.

### Institutional Review Board Statement

The current study was conducted according to the guidelines in the Declaration of Helsinki, and all procedures involving research study participants were approved by the Ethics Committee of the School of Public Health, Shandong University, reference number LL20200801.

### Informed consent

All subjects gave their written informed consent for inclusion before participating in the study.

## Results

Following the systematic methodology, the final version of the knowledge, attitude, and practices (KAP) questionnaire was comprised of four main sections, a total of 76 items: demography (13 items), knowledge (21 items), attitude (20 items), and practice (22 items) (Table 1).

**Table 1:** KAP questionnaire on “oil, salt and sugar”.

Domains	No	Measurements	Response choices
Demography	13	Socio-demographic, level of education, occupation, income level, BMI, past medical history	Open-ended, closed-ended, multiple-choice
Knowledge	21	Etiology, mechanism, nutrition knowledge, RNI	Yes/No/I don’t know, multiple-choice
Attitude	20	Attitudes regarding “oil, salt and sugar”	1 = Strongly disagree,2 = Disagree, 3 = Not sure,4 = Agree, 5 = Strongly agree
Practice	22	“Oil, salt and sugar” practices include a healthy lifestyle, the use of oil, salt and sugar	1 = Always, 2 = often, 3 = Occasional, 4 = Never, 5 = Not applicable

### Socio-demographic profile of study participant

A total of 100 canteen staff participated in this study, with a valid response rate of 100%. Table 2 described the demographic characteristics of the study population. Most participants (66%) were females with a mean age of 40.3 ± 10.5 years old, residing in Rural areas (88%). Most participants (26%) had at least three years of work experience. The percentage of people’s monthly income over 3500 was 47%. A higher proportion of participants’ education was in Junior high school or higher (93%).

**Table 2:** Socio-demographic characteristics of the canteen staff (N = 100).

Variables	Mean (SD)	N (%)
Age, years	40.3 (10.5)	
BMI, kg/m^2^	23.3 (3.4)	
Gender		
Male		34 (34)
Female		66 (66)
Nationality		
Han		98 (98)
Others		2 (2)
Residence		
Urban		12 (12)
Rural		88 (88)
Education		
Primary or below		7 (7)
Junior high school		73 (73)
High school or above		20 (20)
Working experience		
< 3		74 (74)
3 ~		22 (22)
10 ~		4 (4)
Marital status		
Single		15 (15)
Married		85 (85)
Income, RMB/month		
< 2000		18 (18)
2000 ~		35 (35)
3500 ~		24 (24)
5000 ~		23 (23)

### Reliability of the questionnaire

The reliability of the questionnaire was evaluated using Cronbach’s α coefficients for internal consistency and Pearson’s correlation coefficient for test–retest reliability. As shown in Table 3, the Cronbach’s α coefficients of the total questionnaire and Knowledge, Attitude and Practice dimensions, respectively, were 0.822, 0.830, 0.752 and 0.700. The Cronbach’s α coefficients were acceptable for knowledge, attitude and practice. Supplementary Table 1 showed the Pearson’s correlation coefficient for the test–retest reliability of the questionnaire. Correlation coefficients between the two administrations of 0.5 to 0.7 were common, and more than 0.7 can be indicated as very good reliability^19^. Based on Supplementary Table 1, coefficients for the knowledge, attitudes and practice scale were more significant than 0.586 (range: 0.586–1.000), 0.798 (range: 0.798–1.000) and 0.687 (range: 0.687–1.000), respectively, which indicated that each scale of the items had higher correlation. Overall, the correlation coefficient to the questionnaire was 0.969, which suggested that the questionnaire had good reliability.

**Table 3:** The Cronbach’s α coefficients of internal consistency of the questionnaire.

Items	Cronbach’s α	No. of items
Overall	0.822	63
Knowledge	0.830	21
Attitude	0.752	20
Practice	0.700	22

### Validity of the questionnaire

The questionnaire’s content and face validity were established through a satisfactory level of agreement among panelists. The construct validity was established by using exploratory factor analysis. The correlation matrix was used to assess the degree of correlation. The KMO test (0.656) and the Bartlett test of sphericity (Chi-squared, df = 378; *P* < 0.001) (Supplementary Table 2) showed that the data met the criteria required for factor analysis.

As Tables 4 and 5 showed, nine potential factors were identified, with a total of 26 items, which explained 70.923% of the variance. Factor 1 contained eight items on the practice of “oil, salt and sugar”; Factor 2 had three items on the knowledge of “oil, salt and sugar” and health; Factor 3 and Factor 4 both contained three items on the attitudes of “oil, salt and sugar” and health; Factor 5 included two items on attitudes towards the diet of low-sugar and Factor 6 contained two items on practice towards fatty foods and added sugar foods. Factor 7 had two items on salt-related knowledge and attitudes towards “oil, salt and sugar” reductions. Factor 8 contained two items on practice towards a healthy diet, and factor 9 included an item on behavior towards cooking with animal fat.

**Table 4:** Cumulative variance contribution rate and load factor.

Total variance explanation									
Elements	Initial eigenvalues			Extract the load sum of squares			Rotation sums of squared loading		
	Total	Variance (%)	Accumulation (%)	Total	Variance (%)	Accumulation (%)	Total	Variance (%)	Accumulation (%)
1	4.630	16.536	16.536	4.630	16.536	16.536	4.311	15.398	15.398
2	3.869	13.816	30.352	3.869	13.816	30.352	2.486	8.880	24.278
3	2.453	8.762	39.115	2.453	8.762	39.115	2.236	7.985	32.263
4	1.925	6.873	45.988	1.925	6.873	45.988	2.103	7.509	39.772
5	1.669	5.961	51.950	1.669	5.961	51.950	1.924	6.871	46.643
6	1.538	5.494	57.444	1.538	5.494	57.444	1.760	6.286	52.929
7	1.388	4.959	62.403	1.388	4.959	62.403	1.728	6.171	59.101
8	1.236	4.414	66.817	1.236	4.414	66.817	1.711	6.109	65.210
9	1.150	4.106	**70.923**	1.150	4.106	70.923	1.600	5.713	70.923
10	0.901	3.217	74.140						
11	0.731	2.610	76.750						
12	0.698	2.494	79.244						
13	0.655	2.340	81.584						
14	0.638	2.278	83.862						
15	0.558	1.994	85.857						
16	0.530	1.893	87.750						
17	0.464	1.656	89.406						
18	0.419	1.498	90.904						
19	0.388	1.386	92.291						
20	0.361	1.290	93.581						
21	0.343	1.225	94.806						
22	0.274	0.978	95.784						
23	0.269	0.961	96.745						
24	0.243	0.869	97.614						
25	0.215	0.767	98.381						
26	0.183	0.654	99.035						
27	0.172	0.613	99.649						
28	0.098	0.351	100.000						

Extraction method: principal component analysis method.

Significant values are in bold.

**Table 5:** The rotated component matrix.

The rotated component matrix*									
	Elements								
	1	2	3	4	5	6	7	8	9
P7 When you buy packaged food, do you pay attention to the salt/sodium content in the food?	0.810								
P22 When shopping for packaged foods, how often do you choose low or no sugar by reading the Nutrition Facts Labels?	0.794								
P18 How often do you measure added sugars with a scale or other measuring instruments when making Chinese food?	0.750								
P10 When shopping for packaged foods, how often do you read the Nutrition Facts Labels to choose low-in-fat or free of trans fatty acids foods?	0.680								
P19 How often do you measure added sugars with a scale or other measuring instrument when making Western desserts?	0.668								
P20 Do you consciously reduce the intake of high-sugar foods in your daily diet?	0.633								
P2 In the last two months, how often have you used a rationed salt spoon?	0.597								
P13 How often do you use the oil control pot when you cook at home?	0.568								
K10 Which of the following foods are rich in fat?		0.862							
K11 What are the harmful effects of high fat intake on human health?		0.824							
K16 What are the harmful effects of high sugar intake on health?		0.669							
A18 Do you think it is necessary to set a window for “three reductions” dishes?			0.790						
A17 Do you think it is necessary to reduce the use of oil, salt and sugar used in this canteen?			0.659						
A1 Do you think the food in this canteen is salty?			0.467						
A11 What do you think about the sugar intake in daily life?				0.820					
A7 Do you think this canteen must reduce the use of cooking oil/fat?				0.548					
A9 Do you agree to reduce the amount of cooking oil/fat used in the cooking process?				0.492					
A14 Do you agree with the view that we should drink more boiled water instead of sugary drinks?					0.852				
A13 Are you in favor of a low-sugar diet?					0.755				
P11 How often do you eat fatty foods?						0.796			
P15 How often do you eat processed foods with added sugar?						0.684			
K3 What are the harmful effects of excessive salt intake on human health?							0.712		
A19 What difficulties do you think in setting up a "three reductions" window in the canteen?							0.604		
P5 How often do you eat salty foods with high salt content, such as pickled vegetables and salted duck eggs?								0.708	
P14 How often do you drink leftover dishes and soup when you eat at home?								0.679	
P9 How often do you cook with animal fat when you are cooking?									0.806
Extraction method: principal component analysis method									
Rotation method: Caesar normalization maximum variance method									

*The rotation has converged after 15 iterations.

## Discussion

To date, there is no validated KAP questionnaire about the “oil, salt and sugar” used among canteen staff. Therefore, to fill this gap, we established and validated a questionnaire to evaluate the KAP of canteen staff about the “oil, salt and sugar”. This questionnaire is meant to be used by researchers interested in assessing these aspects in the Chinese canteen staff. It is easy to administer and only takes 15 to 20 min to complete for canteen staff.

The final questionnaire comprised three domains (knowledge, attitudes and practice) and 76 items. Canteen staff as participants (N = 100) randomly selected in three college canteens in Shandong Province finished the questionnaire twice to assess reliability and validity. The selected knowledge, attitude and practices scales showed acceptable and satisfactory internal consistencies and good reliability.

Reliability refers to the degree of consistency with which the same results are obtained when the same indicators or measurement tools are used to measure the same thing repeatedly. A questionnaire can be reliable but invalid, but a valid questionnaire is always reliable. Validity is the degree to which a questionnaire evaluates what it is intended to evaluate.

Cronbach’s α coefficient is often used to evaluate the internal consistency of the questionnaire, which is the method employed in most validation studies^20^. Cronbach’s α value higher than 0.67 is almost acceptable, according to Paiva and Sasaki^21^. Values > 0.80 are considered good results for authors like Paiva^20^ or excellent, according to Castro^22^. In our study, although five values were slightly under 0.7 for one construct, Cronbach’s α coefficients of the total questionnaire and each dimension were 0.822, 0.830, 0.752, and 0.700, respectively, which indicated the questionnaire as a whole had adequate internal uniformity and was at a satisfactory level. It was better than a questionnaire about the health literacy scale for low salt consumption^23^. The Pearson’s correlation coefficient of the total questionnaire was 0.968, indicating that the questionnaire had high stability and good test–retest reliability^19^. Furthermore, the “oil, salt and sugar” KAP questionnaire correlated with two more nutritional knowledge questionnaires^24,25^ to assess the test–retest reliability by using a correlation coefficient to demonstrate that the results were consistent over time. Our results seemed to be among the best reported in the literature for the KAP questionnaire about the “oil, salt and sugar” in general, varying from 0.50 to 0.90. These results supported the evidence of temporal stability for the KAP questionnaire.

Validity measures whether the comprehensive evaluation system can accurately reflect the purpose and requirements. Refers to the degree to which a measurement tool can measure the correctness of its intended feature. The higher the validity, the better the measurement results can show the characteristics it is intended to measure. In exploratory factor analysis, nine common factors were extracted, with a total of 26 items, and the cumulative contribution rate was 70.9%; a similar value was obtained in the study in India^26^. This questionnaire was comprehensive and covered the knowledge of oil, salt, sugar, and health and sources of knowledge. Additionally, it covered attitudes toward using these substances in cooking and habits and dietary behaviors to reduce their consumption in daily life. This compensated for the lack of a comprehensive KAP survey on oil, salt, sugar and health in previous studies^27–30^.

This study has several strengths. First, it involved several rounds of group discussion and expert consultation in developing the questionnaire. Second, it had a comprehensive questionnaire composed of several aspects in the knowledge part: the source of knowledge, the relationship between oil or salt or sugar with health, recommended intakes, and identifying unhealthy food with high fat, salt or sugar. The attitude and practice items are based on knowledge items. However, there are limitations related to the study design. Limitations of the study include the small sample size, as many argue that sample sizes of 300 or more are needed for questionnaire development and validation^18^. However, the sample size of this study was considered sufficient and fulfilled the criteria to perform the appropriate statistical tests for the questionnaire validation. Additionally, the self-reported questionnaire was the primary source for collecting the information from the canteen staff, and the quality of the data delivered by the study participants was arguable.

For further studies, this questionnaire can be applied to assess KAP related to using oil, salt, and sugar among canteen staff in other areas of China. However, the questionnaire’s applicability among canteen staff outside China requires further examination. As the participants were Chinese, numerous items were developed according to Chinese guidelines and resources. Thus, additional test validations and modifications are necessary to confirm applicability beyond China.

## Conclusion

A new validated questionnaire was developed for determining KAP about “oil, salt and sugar” among canteen staff. It was shown to have adequate validity and reliability. Thus, it is a valid questionnaire that assesses knowledge, attitudes and practice levels among canteen staff in China about “oil, salt and sugar”.

### Supplementary Information

Supplementary Table 1.

Supplementary Table 2.

## Data Availability

The dataset from the current analysis is not public but is available from the corresponding author upon reasonable request.

## Abbreviations

KAP: Knowledge, attitude and practice
NCDs: Non-communicable chronic diseases
CVDs: Cardiovascular diseases
DALYs: Disability adjusted life years
KMO: Kaiser–Mayer–Olkin
