# Supplementary material for: Reliability and validity of a questionnaire measuring knowledge, attitude and practice regarding “oil, salt and sugar” among canteen staff
Source: Sci Rep. 2023 Nov 22;13:20442. doi: 10.1038/s41598-023-47804-3 (PMC10665314; doi:10.1038/s41598-023-47804-3)
Supplement: Supplementary file 1 — Supplementary Table 1. [file 41598_2023_47804_MOESM1_ESM.docx]

**Supplementary tables 1** The test-retest reliability coefficients of the questionnaire.

| **Items** | **Correlation Coefficient** |
| --- | --- |
| Overall Questionnaire | 0.968 |
| K1 | 0.914 |
| K2 | 1.000 |
| K3 | 0.701 |
| K4 | 0.874 |
| K5 | 0.815 |
| K6 | 0.663 |
| K7 | 1.000 |
| K8 | 0.987 |
| K9 | 0.631 |
| K10 | 0.634 |
| K11 | 0.586 |
| K12 | 0.916 |
| K13 | 0.936 |
| K14 | 0.804 |
| K15 | 0.660 |
| K16 | 0.765 |
| K17 | 0.880 |
| K18 | 0.802 |
| K19 | 0.901 |
| K20 | 0.781 |
| K21 | 0.810 |
| A1 | 0.889 |
| A2 | 0.798 |
| A3 | 0.876 |
| A4 | 0.879 |
| A5 | 0.905 |
| A6 | 1.000 |
| A7 | 0.957 |
| A8 | 0.981 |
| A9 | 0.879 |
| A10 | 0.893 |
| A11 | 1.000 |
| A12 | 0.811 |
| A13 | 0.944 |
| A14 | 0.843 |
| A15 | 0.861 |
| A16 | 0.802 |
| A17 | 0.975 |
| A18 | 0.880 |
| A19 | 0.882 |
| A20 | 0.933 |
| P1 | 0.945 |
| P2 | 0.997 |
| P3 | 0.958 |
| P4 | 0.910 |
| P5 | 0.957 |
| P6 | 0.717 |
| P7 | 0.991 |
| P8 | 0.988 |
| P9 | 0.978 |
| P10 | 0.952 |
| P11 | 0.957 |
| P12 | 0.687 |
| P13 | 0.932 |
| P14 | 0.976 |
| P15 | 0.973 |
| P16 | 1.000 |
| P17 | 0.890 |
| P18 | 0.970 |
| P19 | 0.971 |
| P20 | 0.898 |
| P21 | 0.802 |
| P22 | 0.943 |
