# Supplementary material for: Reliability and validity of a questionnaire measuring knowledge, attitude and practice regarding “oil, salt and sugar” among canteen staff
Source: Sci Rep. 2023 Nov 22;13:20442. doi: 10.1038/s41598-023-47804-3 (PMC10665314; doi:10.1038/s41598-023-47804-3)
Supplement: Supplementary file 2 — Supplementary Table 2. [file 41598_2023_47804_MOESM2_ESM.docx]

**Supplementary Table 2** KMO and Bartlett sphericity test

| **KMO and Bartlett test** | | |
| --- | --- | --- |
| KMO sampling appropriateness quantity | | 0.656 |
| Bartlett sphericity test | chi-squared | 1138.451 |
|  | df | 378 |
|  | Sig. | <0.001 |
